# The Impact of Population Aging on Food Consumption of Rural Households in China: Cross-Sectional Study Across the Ten Geographic Regions

**DOI:** 10.3390/foods15061008

**Published:** 2026-03-12

**Authors:** Tingyu Wang, Dingde Xu, Dong Yang, Mengding Li, Hongxing Lan

**Affiliations:** 1 College of Management, Sichuan Agricultural University, Chengdu 611130, China; wangtingyu1209@163.com (T.W.); dingdexu@sicau.edu.cn (D.X.); 2 Key Laboratory of National Food Security and Tianfu Granary, Sichuan Agricultural University, Chengdu 611130, China; 3 China Rongtong Agricultural Development Group Co., Ltd., Beijing 100036, China; 18971631992@163.com; 4 School of Economics and Management, Hubei University of Arts and Science, Xiangyang 441053, China

**Keywords:** rural households, food consumption, population aging

## Abstract

Food and nutrition are the foundation for human survival. This study focuses on the strategic goals of a Healthy China and actively responding to population aging, empirically examining the impact effects and mechanisms of population aging on the food consumption quantity of rural residents in China. Based on a sample of 2846 rural households from 10 provinces in the China Rural Revitalization Survey (CRRS), this study employs various methods such as Ordinary Least Squares (OLS), instrumental variable methods, and mediation effect models for empirical testing. The study found that population aging has a significant positive impact on the consumption of grains, vegetables, legumes, dairy products, and eggs among rural residents in China, while showing a significant negative impact on the consumption of beef/lamb and fruits. For every 1 percentage point increase in the proportion of individuals aged 60 and above in a household, the consumption of grains, vegetables, legumes, dairy products, and eggs increased by an average of 106.857 g/day, 57.900 g/day, 8.202 g/day, 9.226 g/day, and 7.116 g/day, respectively. The consumption of beef/lamb and fruits decreased by an average of 5.585 g/day and 36.316 g/day, respectively. Although population aging has led to a decrease in the consumption of certain food items among rural residents, the increase in grain consumption has resulted in an enhanced total energy intake per capita for households. Household income levels and the scale of land management play important mediating roles in the impact of population aging on the quantity of food consumed by rural residents. Therefore, corresponding countermeasures are proposed, aiming to promote the construction of a food consumption monitoring system in China, broaden income channels for rural residents, systematically carry out nutrition and health education, and improve the rural social security system in China, thereby optimizing the structure of food consumption.

## 1. Introduction

The problem of nutritional deficiency and excess caused by food consumption is a major problem in the world, especially in developing countries [1]. To promote food consumption improvement, various countries have introduced a series of policies aimed at enhancing food consumption and nutritional improvement, such as the United States Federal Government’s Nutrition Labeling and Education Act, the Dietary Guidelines for Americans (2020–2025), the Food Nutrition Labeling Regulations, Japan’s Nutrition Improvement Law, Denmark’s Fat Tax, and France’s Sugar-Sweetened Beverage Tax [2].

China is facing a severe trend of population aging, which is particularly pronounced in rural areas [3,4]. In 2020, the population aged 60 and above in rural China accounted for 23.81% of the total, while those aged 65 and above made up 17.72%. According to the standards established by the United Nations [5], rural China has now entered an “aged society” and is on the verge of transitioning into a “hyper-aged society” [6]. The proportion of elderly individuals in both societal and family contexts will continue to rise. The deepening of population aging may lead to a restructuring of food consumption patterns among rural residents in China [7]. Against this complex backdrop, it is necessary to gain a more comprehensive understanding of the impact of population aging on the dietary consumption patterns of rural residents to ensure China’s food security.

As the aging population intensifies, the proportion of elderly households in rural areas is rapidly increasing, and the average age of family members is also experiencing an upward trend [8]. This shift in the age structure of household members necessitates a reevaluation of the impacts brought about by population aging at the family level. Considering the intergenerational differences in dietary habits and consumption preferences between the elderly and other age groups, this deepening of household-level aging may lead to a reconstruction of food consumption patterns among rural residents in China [9].

The academic community has conducted a series of discussions on the issue of food consumption related to population aging. Regarding the quantity of food consumption in relation to population aging, existing research mainly presents the following trends. Some studies indicate that population aging generally has a suppressive effect on residents’ food consumption. For instance, Li et al. explored the impact of demographic changes on energy intake based on the 2009 urban household survey data from the National Bureau of Statistics of China, revealing characteristics of energy intake changes across the lifecycle of Chinese residents. The research found that changes in population structure significantly affect the energy intake of urban households in China, with the energy intake of individuals aged 60 and above showing a trend of initially increasing and then decreasing, particularly after the age of 65. This suggests that aging will lead to a decline in food consumption in China [9]. Gao M et al. compared the trends in grain consumption of urban and rural residents between 1985 and 2020 using statistical yearbook data and nationally representative household data from fixed rural observation points in China. Their findings show that an increase in the elderly population within households leads to a decrease in the absolute consumption of all types of food [10]. The underlying reason is that this common conclusion of declining total consumption is largely derived from physiological degeneration and life-cycle theory, namely that the reduction in elderly individuals’ basal metabolic rate leads to lower caloric requirements.

In another study, some research argues that the impact of population aging on food consumption exhibits significant heterogeneity. For example, Wen et al. found that young farmers in China have significantly stronger consumption habits for grains, vegetables, fruits, pork, eggs, and cooking oils compared to the middle-aged and elderly populations, while the latter group shows more stable consumption patterns for legumes and aquatic products [11]. Additionally, Deng et al. discovered that the consumption of staple foods and basic life staples among the elderly is significantly lower than that of other age groups; however, their consumption of nutritious staples (such as dairy products) is markedly higher, indicating significant differences in food consumption structure within different age groups, as well as persistent disparities in food consumption between urban and rural populations across age groups [12]. The divergence in these research findings stems, on the one hand, from differences in research dimensions—such as whether the focus is on energy intake or specific food categories—and, on the other hand, from changes in the broader socio-economic context. In addition, the impact of the urban–rural dual structure is also an important source of variation: asymmetries between urban and rural elderly in terms of access to food and purchasing power cause the general pattern of total consumption to exhibit more diversified characteristics when examined in segmented samples.

Furthermore, scholars have deeply explored the mechanisms by which aging influences food price elasticity and preferences for nutrients. For instance, Han et al. utilized US data and, through a demand system model, found that the income elasticity of demand for fats and animal products was 0.53 and 0.67, respectively, for elderly individuals living alone or with a spouse, which was lower than that of groups with a lower proportion of working-age individuals (0.55 and 0.68). More significantly, the income elasticity of demand for fruits and vegetables for the elderly (0.79) was higher than in the group with a low proportion of working-age individuals (0.76), implying that the elderly are more sensitive to changes in consumption of fruits and vegetables with changes in income. Therefore, in the context of escalating population aging, ensuring the supply of fruits and vegetables becomes particularly crucial to meet the growing demands of the elderly population [13]. The study by Nuno Mendonca et al. further confirmed the close association between age structure and food consumption, indicating that aging systematically alters people’s demand and preferences for nutrients such as protein, fat, and carbohydrates, thereby influencing overall dietary levels [14].

Concurrently, in an agricultural context, farm size and household income level are influential drivers. Farm size generally has a positive impact on dietary diversity; a larger farm size directly enhances a household’s ability to produce multiple food crops, leading to a wider variety of dietary choices [15,16]. For example, Koppmaire et al. used survey data from smallholder farmers in central and southern Malawi and found a positive relationship between agricultural production diversity and dietary diversity [17]. Consistent with the findings of Romeo A et al. and Sibhatu et al., they argued that for rural residents in resource-limited and impoverished regions, diversified agricultural production for self-sufficiency can increase the availability of various foods, thus ensuring dietary diversity for the household [18,19]. In addition to this, household income level is another key determinant of food consumption. As income increases, household purchasing power strengthens, enabling them to access a wider variety of foods beyond basic staples, including more nutrient-dense animal products, fruits, vegetables, and legumes, which are typically more expensive than grain-based staples [20]. Multiple studies have confirmed the correlation between household income and food consumption patterns [21,22]. For instance, Teshome K. et al., through a study of 1200 households in nine districts of Ethiopia, found that higher income was significantly correlated with increased dietary diversity [23]. Chen Y. et al.’s research also indicated that income growth promotes a shift in household diets from grain-centric to protein-rich foods and fresh produce [24]. Nevertheless, the impact of income is also influenced by factors such as income source, stability, and local food prices and availability, and expenditure patterns of income are equally important; high income does not necessarily guarantee healthier dietary choices, especially in the absence of nutritional knowledge.

In summary, although existing studies have extensively examined the impact of population aging on food consumption, they still suffer from the following limitations: First, in terms of analytical dimension, existing studies mostly focus on the individual level and overlook the structural evolution of the household, which is the basic unit of consumption decision-making. Although prior research has shown that elderly individuals reduce their energy intake due to physiological decline, in rural China, intergenerational support, co-residence, and other forms of family interaction often reshape household consumption decisions. When the average age of household members rises or the proportion of elderly members increases, how the household’s overall dietary structure shifts remains largely unexplored in systematic empirical work. Second, in terms of geographical and economic context, research specifically targeting rural areas is notably insufficient. Many studies either pool urban and rural data or focus on nutritional transitions among urban residents. Because rural residents differ systematically from urban residents in income elasticity, access to food, and health awareness, urban-based models cannot accurately capture dietary restructuring under rural population aging. In particular, against the backdrop of continued out-migration of young and middle-aged labor from rural areas, household aging in the countryside reflects not only biological characteristics, but also a profound mismatch between production and consumption patterns; its implications for food demand call for more in-depth investigation. Finally, in terms of methodology and research perspective, most existing studies concentrate on a single food category or on total energy intake, and lack a comprehensive examination of multi-category food demand systems. Some studies discuss age-specific preferences for particular foods, but have not systematically explained how aging reshapes food consumption structures at the household level.

In this context, this paper uses microdata from the China Rural Revitalization Survey to examine the impact of household aging on dietary consumption in rural China. We not only analyze the effects of aging on the consumption of different food categories and on total dietary energy intake, but also explore the underlying mechanisms from the perspectives of “purchasing capacity” and “self-supply capacity,” drawing on the absolute income hypothesis and the sustainable livelihoods capital framework, respectively. The findings aim to provide empirical evidence for addressing rural aging, improving the nutritional status of rural residents, and formulating targeted food security policies.

## 2. Theoretical Analysis and Research Hypothesis

### 2.1. The Direct Impact of Population Aging on Food Consumption Quantity Among Rural Households

The rapid increase in the proportion of elderly people in rural China profoundly affects the food consumption patterns of rural households [10]. As people age, their physical functions decline, and their food and nutrient needs decrease [25]. Furthermore, with the large-scale out-migration of young labor, rural population aging exhibits distinct characteristics of “unnatural aging.” This results in an “aging-led” phenomenon, primarily driven by labor out-migration rather than natural life expectancy extension [26,27]. The food consumption characteristics of the elderly have become an important representation of the food consumption patterns of rural households, indicating that population aging will have a series of impacts on the quantity of food consumption among rural residents.

Firstly, population aging modifies the aggregate food consumption of rural households. With advancing age, physiological changes occur, including reduced metabolism, blunted taste and smell, weakened digestion, and impaired oral function, generally resulting in lower food intake among the elderly [28,29,30]. A higher proportion of older adults in rural households implies a reduced share of high-energy-demand individuals, thereby lowering both average household energy requirements and overall energy intake from food.

Secondly, population aging also reshapes the food consumption structure within rural households. The dietary preferences of rural elderly individuals, shaped by a combination of traditional customs, economic status, and physiological demands, tend towards seasonal produce, soft and digestible foods, and high-carbohydrate options [31]. Their typical consumption pattern is characterized by a predominance of staples (such as grains and legumes), supplemented by seasonal vegetables, eggs, and dairy products, with relatively limited intake of pork, poultry, and beef/lamb. As the proportion of the elderly population increases, these dietary patterns are amplified at the household level, potentially leading to a significant increase in staple consumption, stable or slightly increased intake of vegetables, dairy products, and eggs, largely unchanged or slightly decreased intake of pork, poultry, and beef/lamb, and a noticeable reduction in fruit consumption.

In summary, this paper proposes the following hypotheses:

**Hypothesis** **H1:** *Population aging has a significant negative impact on the total energy consumption of food among rural households.*

**Hypothesis** **H2:** *The impact of population aging on the consumption of different types of food among rural households varies.*

### 2.2. The Indirect Impact of Population Aging on Food Consumption Quantity Among Rural Households

Food consumption in rural areas differs from that in urban areas, encompassing not only the market supply channel for food purchases but also self-supply of agricultural products, which becomes an important pathway through which the aging population affects the food consumption quantity of residents. This section will systematically explore the intermediary roles of household income levels and land management scale in the impact of population aging on food consumption quantity among rural residents from the perspectives of self-sufficiency in food and purchasing ability.

#### 2.2.1. The Mediating Role of Household Income Level

Based on sustainable capital theory, the changes in household human capital caused by population aging will lead to shifts in livelihood strategies and outcomes. Population aging not only exacerbates income disparities between urban and rural areas but also leads to income inequality within rural areas [32]. In rural areas, where physical labor is predominant and families tend to be older, income levels are generally lower. Income is a prerequisite for consumption [33], and the aging of household populations may lead to a decrease in rural household income levels, subsequently affecting their consumption capability and ultimately influencing the quantity of food consumed by rural residents. Specifically, population aging will significantly lower household income levels. On one hand, regarding the impact of population aging on household agricultural income, aging affects rural households’ human capital. With the decline in physical strength among elderly laborers, there is likely a preference for growing low-labor-intensity crops, reducing cultivated land area, and lowering the cropping index, which leads to decreased agricultural output and income [34]. On the other hand, in terms of the impact on household non-agricultural income, elderly individuals have a significantly reduced ability to obtain higher-paying non-agricultural employment, primarily relying on local low-paid labor or pensions, which leads to an overall reduction in wage income for the household [35].

Household income levels further influence the quantity of food consumption. The absolute income hypothesis theory emphasizes that “income determines consumption”; thus, the decline in income due to the aging of rural household populations may diminish their capacity for food consumption [36]. However, it should be noted that due to food consumption elasticity, the impact of this consumption capability on food consumption behavior may vary across different types of food, having a stronger suppressive effect on the consumption of nutritional side dishes while potentially having less impact, or even a positive effect, on the consumption of staple foods such as grains. Of course, it is important to consider the preferences of elderly individuals regarding ingredients, cooking methods, and dietary habits during this impact process. Overall, population aging will lower the per capita income level of rural households, thereby inhibiting the total energy intake from food. Therefore, the following hypothesis is proposed:

**Hypothesis** **H3:** *Household income level mediates the impact of population aging on the quantity of food consumption among rural residents.*

#### 2.2.2. The Mediating Role of Land Management Scale

Agricultural family management is an organizational form in which farming families independently or relatively independently engage in agricultural production and management activities [37]. As the basic management system in rural China, it utilizes social services and advanced technologies to break through the limitations of small-scale farming economies and forms the foundation of a new agricultural management system. However, with the continuous evolution of population aging, the scale of land management for rural families is affected. First, population aging places constraints on the labor force for rural household land management. Elderly laborers experience physical decline and find it difficult to undertake large-scale and high-intensity farming; thus, rural elderly families tend to sublet or fallow some of their land, proactively reducing their management scale [38]. Second, population aging leads to a shift in the management goals of rural families. Affected by aging, elderly households tend to shift from pursuing maximum output to satisfying their own food needs or low-intensity management, resulting in a reduced willingness to engage in large-scale operations.

**Figure 1:**
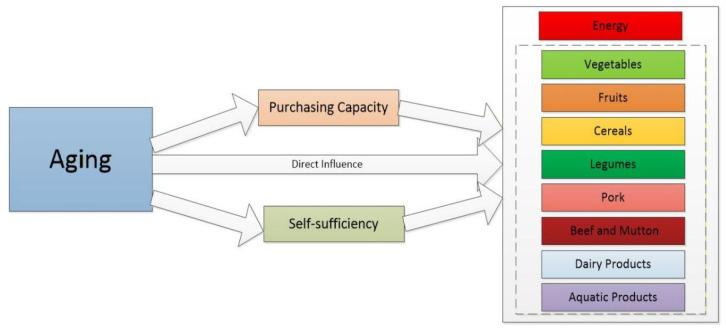
Mechanism of the Impact of Population Aging on Food Consumption among Rural Residents.

Land management is not only a significant avenue for economic income for rural families but also an important source of food acquisition. The decline in household land management scale brought about by population aging may further lead to changes in food consumption quantity. On one hand, the scale of management directly determines the quantity of food available from self-production. In rural areas, production and management decisions are typically made at the household level, and during the production process, food items such as grains, vegetables, and fruits are often grown for “self-use,” which becomes an essential source of food consumption [39]. The quantity of consumption of various foods largely depends on whether households engage in planting and the scale of cultivation. On the other hand, managed scale is an important means of increasing agricultural income; the reduction in scale caused by aging will decrease agricultural cash income, thereby affecting the purchasing power for commercially sourced food (as shown in Figure 1). Therefore, the following hypothesis is proposed:

**Hypothesis** **H4:** *Land management scale mediates the impact of population aging on the quantity of food consumption among rural residents.*

## 3. Methods

### 3.1. Data Sources

The micro-survey data used in this study originates from the “China Rural Revitalization Survey” (CRRS) conducted by the Institute of Rural Development at the Chinese Academy of Social Sciences. The CRRS data adopts a strict sampling scheme that follows the principle of random stratified sampling, covering 10 provinces, 50 counties (cities and districts), 150 townships, and 300 administrative villages across China.

First, the project team took into account multiple factors such as economic development levels and geographic locations, selecting a total of 10 provinces: Zhejiang, Guangdong, Shandong, Henan, Anhui, Guizhou, Sichuan, Shaanxi, Ningxia, and Heilongjiang, in proportion to one-third of the number of provinces in each region (East, Central, West, and Northeast China), Second, considering the spatial uniform distribution of counties, the counties (cities and districts) in each province (region) were divided into five groups based on per capita GDP levels. One county was randomly selected from each group, resulting in five counties being chosen from each province. Then, following a similar principle to that used for county selection, three townships were randomly selected from each county, representing high, medium, and low economic development levels. Subsequently, two administrative villages representing high and low economic development levels were randomly selected from each township. Finally, based on the rosters provided by each village committee, approximately 10 households were selected using an interval sampling method from each administrative village, and questionnaire surveys were conducted regarding household population, labor employment, income, and consumption. After excluding observations with key missing values and outliers, the final sample size is 2846. Verbal informed consent was obtained from the participants. Verbal consent was obtained rather than written because the respondents in this survey are predominantly rural residents, some of whom have limited literacy. Furthermore, there is often a natural psychological reluctance within rural society towards signing formal written agreements. To mitigate respondents’ apprehension, ensure the authenticity of the data, and strictly adhere to anonymity principles, the research team decided to inform participants orally and obtain their verbal consent.

### 3.2. Model Setting

#### 3.2.1. Baseline Regression Model

The data used in this study is cross-sectional data, and since the dependent variable, food consumption quantity, is a continuous variable, the model employs the Ordinary Least Squares (OLS) regression.

Formula (1) primarily aims to examine the impact of population aging on the food consumption quantity of rural households. In the equation, i represents rural households, Quantity_i_ denotes the continuous dependent variable, and Aging_i_ serves as the core explanatory variable, capturing the variable for population aging, while X_i_ includes a set of control variables. The household characteristics and rural attributes are also included. ε_i_ is the error term, β_1_ and β_i_ are the estimated parameters. The specific model is set as follows:

(1) Quantity_i_ = β_0_ + β_1_ Aging_i_ + … + β_i_X_i_ + ε_i_

#### 3.2.2. Mediation Effect Model

This study employs a mediation effect testing method to empirically validate the mechanism through which population aging affects the food consumption quantity of rural households. Considering that the causal relationship between the selected mediating variables and the dependent variable, food consumption quantity, is relatively clear and intuitive, this study focuses on the impact of the core explanatory variable of population aging on the mediating variables, referencing Jiang Ting’s recommendations on mediation effect analysis in causal inference studies [40]. The econometric model is specified as follows:

(2) Mediator_i_ = θ_0_ + θ_1_ Aging_i_ + … + β_i_X_i_ + μ_i_ + ε_i_

In the equation, the subscript i represents the rural household, Mediator_i_ denotes the mediating variable reflecting the impact of population aging on rural household food consumption quantity, which includes household income levels and land management scale. Aging_i_ is the core explanatory variable representing population aging. X_i_ consists of a series of control variables, primarily including household characteristics and community attributes. μ_i_ is the district fixed effect, ε_i_ is the error term, β_i_ and θ_1_ are estimated parameters.

### 3.3. Main Variables and Descriptive Statistics

#### 3.3.1. Explained Variable

Measurement standards in academia. These primarily include whether a household has elderly members. The CRRS employs a 30-day recall method to calculate household food consumption and expenditure, targeting household members who regularly share meals. To facilitate calculations and comparisons, monthly household consumption data is converted into daily average household consumption data for analysis. When analyzing food consumption, this study uses the food classification standards from the “Chinese Food Composition Table.” It aggregates the original 26 detailed food categories into broader categories. For the sake of analysis, this chapter does not report on food items such as tobacco, alcohol, tea, beverages, and condiments, focusing instead on ten categories of food: grains, fruits, vegetables, legumes, meat (including beef, lamb, and pork), poultry, dairy products, eggs, and aquatic products.

This study considers the household as the basic unit. Among the 2846 households surveyed, the average number of household members was 3.307. The minimum household size was 1 person, and the maximum was 15 people. Due to differences in age and sex, individuals have varying food intake requirements. Using per capita consumption to represent the food consumption and energy intake at the household level overlooks the compositional differences within households. Existing studies typically use a standard person of a 60 kg adult male engaged in light physical activity (with a caloric intake of 2250 kcal). Based on the “Chinese Dietary Reference Intakes 2013 (DRIs),” recommendations for nutrient requirements vary by age, gender, and activity level, allowing for the calculation of standard person coefficients (the ratio of nutrient requirements to the standard person’s nutrient requirements) for different types of residents, which are then summed to determine the household’s standard person count.

This study focuses on the rural population, with agricultural producers accounting for a significant proportion of the sample, at 64.86%. First, rural residents primarily engage in agricultural production activities such as farming and animal husbandry, with generally higher labor intensity compared to sedentary urban populations, as agricultural work typically involves moderate physical labor. Second, aside from productive labor, rural residents also undertake household chores of significant intensity, with walking being the primary mode of transportation, further increasing daily total energy expenditure. Establishing a moderate labor intensity standard more accurately reflects the real energy needs of rural households, avoiding biases in nutritional interventions or health recommendations due to underestimating labor intensity. Therefore, this study uses a standard person of a 60 kg adult male engaged in moderate physical activity (with a caloric intake of 2550 kcal) and, based on the latest “Chinese Dietary Reference Intakes 2023 (DRIs),” calculates the standard person coefficients for different types of residents according to their nutrient requirements, summing to conclude the household standard person count.

In this study, the dependent variable is food consumption quantity, represented by the energy intake (kcal) per household standard person. Specifically, regarding food types, food consumption quantity includes the amounts of grains, fruits, vegetables, legumes, beef, lamb, pork, poultry, dairy products, eggs, and aquatic products per household standard person.

#### 3.3.2. Key Variables

Regarding the measurement of population aging within households, there are various measures, such as the absolute number of elderly individuals and the proportion of elderly individuals within the household [41,42,43]. The proportion of elderly members in the household is more intuitive and better reflects the concept of population aging compared to simply determining whether there are elderly individuals in the household, making it more widely used.

In studies that measure household population aging using the proportion of elderly members, researchers often use the percentage of household members aged 60 and above or 65 and above as the benchmark [42]. This study defines elderly individuals based on Article 2 of the “Law of the People’s Republic of China on the Protection of the Rights and Interests of the Elderly,” which took effect on 1 July 2013, stating that elderly individuals are citizens aged 60 and above. This study uses the proportion of elderly individuals aged 60 and above relative to the total household population to represent the degree of population aging in the household.

#### 3.3.3. Control Variables

Rural households’ decisions regarding food consumption quantities are influenced by various factors. This study incorporates control variables from three main aspects: head of household characteristics (such as gender, ethnicity, education level, and marital status), household characteristics (including political affiliation, female proportion, participation in medical insurance, and participation in pension insurance), and village characteristics (like the distance to the county government, the village’s economic level, terrain, and whether the village is part of an urban suburb).

## 4. Results

### 4.1. Baseline Regression Estimates

As shown in Table 1, the measurement standard for food consumption quantity in this study is the household standard per capita energy intake and the daily average consumption in grams of various food types per household. Since this is a continuous variable, Ordinary Least Squares (OLS) is used for model estimation. Table 2 presents the baseline regression results of the impact of population aging on the total energy consumption and various food consumption quantities of rural households. This result does not consider endogeneity issues but still allows for a preliminary analysis of the effects of population aging on food consumption in rural households. Column (1) provides the estimated result of the impact of population aging on the household standard per capita total energy intake, while columns (2) to (11) present the estimated effects of population aging on daily per capita consumption quantities of grains, vegetables, fruits, legumes, dairy products, pork, poultry, beef, lamb, eggs, and seafood.

The regression results indicate that population aging exerts a significantly positive effect on per capita household standard total energy intake at the 1% level, The marginal effect indicates that after controlling other factors, the average daily total energy intake increases by 448.997 kcal per 1 percentage point increase in the proportion of household members aged 60 and above, which is equivalent to 14.075% of the average daily energy intake of rural residents in the sample, contradicting the research hypothesis H1. A plausible explanation is that the primary channel through which aging increases per capita energy intake is not an augmentation in other food categories but a notable rise in cereal consumption. Table 2, which documents the impact of aging on rural households’ consumption across various food categories, shows that aging markedly increases cereal consumption. Cereal, as the principal energy source for Chinese households, possesses relatively high energy density. According to the China Food Composition Table, 100 g of rice (a representative staple) provides 346 kcal. In our sample, the average daily per capita cereal intake is 491.618 g, implying an average energy intake from cereals of approximately 1700 kcal per person per day, accounting for 53.29% of total energy intake. Thus, even if aging reduces the consumption of certain foods, the substantial increase in cereal consumption—driven by its high energy density—can offset, or even exceed, the energy reductions from other foods, leading to an overall rise in per capita total energy intake. This finding suggests that, when examining the impact of population aging on residents’ healthy eating patterns, it is necessary to scrutinize changes across food categories and their combined effects on total energy intake more carefully.

Concurrently, Table 2 reveals that aging exerts a significantly positive effect at the 1% level on per-household consumption standards for cereals, vegetables, legumes, and eggs, and a marginally significant positive effect at the 10% level on dairy products. In contrast, the effects on per capita fruit consumption and on beef and mutton consumption are significantly negative at the 1% level. These results confirm the existence of heterogeneous effects of aging on different food categories within rural households. Specifically, controlling for other factors, a 1 percentage point increase in the share of the population aged 60 and above is associated with an average increase in cereal consumption by 106.857 g/day, vegetables by 57.900 g/day, legumes by 8.202 g/day, dairy products by 9.226 g/day, and eggs by 7.116 g/day. Aging also exerts a significant suppressive effect on certain high-value foods: a 1 percentage point rise in elderly share is associated with declines in beef and mutton by 5.585 g/day and in fruit by 36.316 g/day, respectively, thereby supporting hypothesis H2. The proposed mechanisms are as follows.

First, from a perspective of dietary preferences and physiological adaptation, the higher intake of cereals, legumes, dairy, and eggs among older adults may reflect physiological changes characteristic of this demographic group. Cereals and dairy, as traditional energy sources and plant protein sources, are readily accessible in daily diets, while eggs—owing to their simple preparation and easy digestibility—have become an important source of animal protein for rural elderly individuals. By contrast, meat, such as beef and mutton, possesses coarser fibrous textures and a greater digestive burden, which may lead to lower intake, illustrating a self-regulation mechanism whereby older individuals adjust their diets to their digestive capacity. Second, from an economic cost and accessibility standpoint, rural elderly individuals exhibit a clear “pragmatic” consumption pattern. Cereals and seasonal vegetables are typically cheaper and often procured within the rural household’s “home economy” of self-sufficiency. In contrast, fruits and beef/mutton are relatively expensive per unit and exhibit higher income elasticity; when income prospects are uncertain, elderly households may reduce these higher-cost foods to optimize budget allocation. This pattern—centering on cereals, vegetables, eggs, legumes, and dairy as core protein sources—can meet basic energy needs but leaves room for dietary balance improvements.

Regarding control variables, the head of household’s ethnic characteristics significantly influence consumption for most food types. Specifically, ethnicity negatively affects per capita cereal and beef/mutton consumption at the 5% and 1% levels, respectively, while positively affecting per capita pork, poultry, eggs, and vegetables consumption at the 1%, 5%, 5%, and 10% levels, respectively. This may reflect divergent dietary cultures and religious beliefs across ethnic groups, which shape consumption preferences and taboos, leading to a richer dietary mix among Han Chinese compared with minority groups whose diets center more on beef and mutton.

The education level of the household head substantially influences most food categories. Specifically, higher education is associated with a negative effect on per capita cereal consumption at the 1% level, and a positive effect on per capita fruit, dairy, and seafood consumption at the 1% and 5% levels. This suggests that greater educational attainment correlates with a shift away from high-carbohydrate foods toward higher-quality proteins and vitamins.

Political identity exhibits differential effects across food categories. Specifically, at the 5% level, political identity negatively affects per capita cereal consumption, while at the 1% and 5% levels, it positively affects beef, mutton, pork, and seafood consumption. Party members display lower per capita cereal consumption but higher consumption of beef, mutton, pork, and seafood. This may reflect that political identity is associated with broader information access, enabling quicker adoption of healthy eating concepts, and with higher socioeconomic status and more stable household income, which supports greater meat consumption.

Pension insurance exerts a significantly positive impact on consumption across multiple food groups. Specifically, pension insurance positively affects per capita cereal, vegetables, and pork consumption at the 1% level, and positively affects per capita poultry and eggs consumption at the 10% level. This indicates that higher pension participation amplifies the consumption of cereals, vegetables, pork, poultry, and eggs. The mechanism extends beyond income transfers: pensions reduce future uncertainty and reshape rural elderly individuals’ “mental accounting.” For them, pension is viewed as “survival funds,” and budget allocations prioritize stabilizing core food consumption (cereals and pork), thereby freeing additional consumption capacity for basic sustenance. However, within traditional rural belief systems, fruits and beef are often regarded as “non-essential” or seasonal items. Even with looser budgets, precautionary saving motives remain strong, so the marginal effect of pension on these “improver” foods is not significant.

Village terrain characteristics significantly affect rural households’ food consumption. Specifically, terrain exerts a positive effect on per capita pork and cereal consumption at the 1% and 5% levels, but a negative effect on per capita poultry, beef, mutton, eggs, aquatic products, and fruit consumption. This implies that households in mountainous and hilly areas consume more pork and cereals than those in plains, while consuming considerably less poultry, beef, mutton, eggs, aquatic products, and fruits. This pattern reflects not only the logistical and supply constraints imposed by geographic conditions but also the path dependence of dietary patterns shaped by long-standing livelihoods. In mountainous and hilly regions, labor-intensive agriculture and material scarcity foster a preference for “high-energy, easy-to-store” foods, such as readily preservable pork and storable staples (cereals). With population aging, these diet habits, formed under specific environmental conditions, exhibit strong inertia and rigidity. By contrast, perishable and high-priced foods (e.g., fruits and aquatic products) face higher market premiums in mountainous areas and conflict with the elderly’s long-standing logic of “minimizing energy acquisition costs,” and are thus less likely to appear in their daily core diet. This territorial effect essentially results from the combined influence of physical environmental constraints and long-term dietary culture accumulation.

### 4.2. Discussion of Endogeneity Issues

The previous section conducted a preliminary analysis of the impact of population aging on food consumption quantities in rural households using a baseline regression model. However, it is important to consider that the model estimates may be subject to endogeneity issues in three main areas. First, omitted variable bias may cause the explanatory variables to be correlated with the error term, leading to inconsistent estimation results. Numerous factors influence residents’ food consumption, such as seasonality, fluctuations in food prices, food markets, dietary knowledge, and eating habits. Since these variables are difficult to capture comprehensively and many are unobservable, omitted variable problems are likely to arise. Second, there may be reverse causality in the model. While household demographic structures tend to be relatively stable, they can still be affected by the migration of labor into and out of the household. Although increasing population aging may impact food consumption, some rural households or individuals within them may choose to seek work elsewhere or migrate to enhance their livelihoods and access a more diverse food supply. Migration of labor in and out of rural households may lead to inconsistent model estimates. Third, there may be systemic biases in the data concerning the proportion of elderly individuals in households or food consumption.

To address potential endogeneity, this study further employs instrumental variables (IV) and conducts endogeneity testing. We use the average aging level of other households in the same village as the instrument and apply two-stage least squares (2SLS) estimation. On one hand, households within the same village share similar social networks and cultural backgrounds; through peer effects or social norms, the aging level of other households can influence the target household’s expectations about its own demographic structure and lifestyle, thereby satisfying the relevance condition. On the other hand, in the current rural context, food supply is primarily determined by regional commerce and logistics systems, which are not easily affected by the population structure of a single village. Moreover, community support systems play a limited role in rural food provisioning and are unlikely to directly affect a household’s total food intake. Thus, the instrument largely satisfies the exogeneity assumption.

Table 3 presents the IV regression results with food consumption as the dependent variable. First, regarding the endogeneity test, the Durbin–Wu–Hausman test yields a *p*-value of 0.270. From a statistical rigor standpoint, this result fails to reject the null hypothesis that population aging is exogenous, indicating that endogeneity biases in the sample may not be severe and that the baseline OLS estimates are reasonably reliable. Nevertheless, given that the power of the endogeneity test can be affected by sample distribution and a theoretical possibility of reverse causality and omitted variables exists, we nonetheless treat the 2SLS estimates as the reference.

The first-stage regression shows a significant positive relationship between the instrument and population aging, with an F-statistic exceeding 10, indicating that weak instrument concerns are unlikely. The second-stage regression indicates that, after addressing endogeneity in the regressors, population aging exerts a significantly positive effect on per capita total energy intake in rural households at the 5% level. The IV estimates’ signs and significance are highly consistent with the OLS estimates, reinforcing the robustness of the conclusion and suggesting that even under relaxed exogeneity assumptions, aging continues to have a positive effect on energy intake among rural residents.

### 4.3. Robustness Check

To further validate the reliability and effectiveness of the above analysis results, this article primarily conducts robustness checks by replacing the core explanatory variables.

As medical technology advances and living standards improve, people’s lifespans are generally extended. The World Health Organization (WHO) has adjusted its age standard for the elderly, raising it from 60 years in 1950 to 65 years. In the baseline model, this study defines elderly individuals as those aged 60 and older, measuring household aging by the proportion of individuals aged 60 and above relative to the total household population. For the robustness check, the age standard for the elderly is increased to 65 years, measuring household aging by the proportion of individuals aged 65 and older relative to the total household population.

After replacing the core explanatory variable, the impact of population aging on household standard per capita total energy intake remains positively significant. Furthermore, the effects of population aging on the consumption of different types of food in rural households continue to exhibit variability. As shown in Table 4, after replacing the core explanatory variable with the proportion of the population aged 65 and older in the household, the impact of population aging on household standard per capita total energy intake is positively significant at the 1% level. This indicates that population aging promotes an increase in household standard per capita total energy intake; the higher the degree of population aging, the greater the household standard per capita total energy intake.

The consumption of various foods shows that population aging has a significant positive impact on the consumption of cereals, vegetables, legumes, dairy products, and eggs. In contrast, its impact on fruits, beef, and mutton is significantly negative, indicating that the effect of population aging on rural households’ food consumption varies across different food categories. This conclusion is highly consistent with previous studies, thereby confirming the robustness and reliability of the impact of population aging on rural residents’ food consumption.

### 4.4. Mediating Effects Analysis

This study has systematically verified the impact of population aging on the food consumption quantities of rural households through baseline regression estimates, endogeneity discussions, robustness checks, and heterogeneity analyses. However, understanding the mechanisms underlying this impact requires further exploration. This section investigates the mediating role of household income levels and land management scale in the relationship between population aging and food consumption quantities in rural households.

Table 5 (1) shows the estimation results based on the OLS model, indicating a significantly negative coefficient for population aging, which suggests that an increase in population aging reduces household income levels. Referencing the studies by Jiang Ting [31], it can be concluded that the intensification of population aging weakens food consumption quantities among rural residents by lowering household income levels, thus validating research hypothesis H3. A possible explanation is that both agricultural and non-agricultural labor in rural areas require a certain level of physical strength. Population aging affects the human capital of rural households; the decline in elderly labor capacity often leads to reduced agricultural and non-agricultural income. The absolute income hypothesis emphasizes that “income determines consumption,” making income capacity a key determinant of consumption ability. Thus, the decline in income resulting from population aging reduces the food consumption capacity of rural households, ultimately manifested as a decrease in total food consumption expenditures, even though grain consumption increases contribute to an overall rise in energy intake.

The study uses the actual area of land operated by households as a proxy for land management scale and conducts regression analysis with it as the dependent variable. Table 5 (2) presents the estimation results from the OLS model, revealing a significantly negative coefficient for population aging, indicating that an increase in population aging reduces the area of land operated by households. This finding suggests that population aging weakens food consumption quantities among rural residents by decreasing the area of land managed, thus validating research hypothesis H4. A possible explanation is that population aging imposes labor constraints on the land management scale in rural households. As elderly laborers experience declines in physical capacity, they find it challenging to undertake extensive and intensive land cultivation. Consequently, elderly households in rural areas are inclined to sublet or abandon portions of their farmland, proactively reducing their operational scale.

Land management is not only an important avenue for economic income for rural households but also a crucial source of food acquisition. A decrease in the scale of land management due to population aging may further lead to changes in household food consumption quantities. Decisions regarding agricultural production and management in rural areas are typically made at the household level; during the production process, crops such as grains, vegetables, and fruits often serve as significant sources of food consumption in the form of “self-retention.” The quantity of food consumed largely depends on whether the household engages in cultivation and the scale of that cultivation.

## 5. Discussion

Overall, existing research has conducted a series of discussions on the causes, trends, influences, and current characteristics of food consumption, as well as the influencing factors and comprehensive effects, thereby laying a solid theoretical foundation and methodological support for future studies. However, there are certain inadequacies in the depth of existing research at the micro level, and further deepening and expansion are urgently needed. First, there is a lack of national microdata. Second, current studies on food consumption among residents mostly focus on the national level or urban areas, with relatively few studies on food consumption among rural residents. Finally, the intrinsic relationship between population aging and residents’ food consumption has not been systematically and sufficiently explored. Therefore, this study utilizes representative national micro-sample data to conduct a focused investigation on the impact of population aging on the household food consumption of rural residents. It aims to clarify the transmission mechanisms through multiple channels, such as purchasing capacity and self-sufficiency, that influence household food consumption among rural residents. The goal is to provide decision-making ideas and policy references for optimizing and upgrading food consumption and nutritional health among rural residents in the context of actively responding to population aging in China.

This article argues that population aging has a significant positive impact on the standard per capita energy intake of rural households and exhibits significant differences in its effect on per capita daily consumption of various food categories. This is similar to the conclusions drawn by Wen J et al. and Deng Tinghe, although the specific types differ. The article maintains that population aging has a significantly positive effect on the consumption of vegetables, legumes, eggs, and grains among rural residents in China, while it has a significantly negative effect on the consumption of fruits and mutton/beef. Unlike the findings of Wen et al., who show that population aging drives higher consumption of health-related high-protein foods, this study finds that rural older adults increase their intake of more economical legume-based protein (plant protein) rather than beef and mutton (high-quality animal protein). This reveals characteristics of “consumption downgrading” and “survival strategies” under the influence of rural aging. In the context of an underdeveloped rural social security system, older people tend to cope with the “liquidity constraints” of longevity and future uncertainty by consuming low-cost subsistence resources (grains and vegetables). The heterogeneous results also reflect the distinctive production and livelihood patterns in rural areas. The positive effect on vegetables and grains may stem from the fact that, after “withdrawing” from heavy agricultural labor, older rural residents shift to cultivating small household vegetable plots near their homes. Maintaining this self-sufficiency reduces the acquisition cost of these foods, whereas fruits and beef/mutton rely heavily on market supply and are highly price-elastic. The decline in labor capacity and the downward adjustment of income expectations caused by aging lead to a marked contraction in such high-priced consumption items. In addition, the study finds that household income level and land management scale play important mediating roles in the impact of population aging on rural residents’ food consumption. The mediating effect of household income confirms that population aging weakens household labor supply, tightens the budget constraint, and forces a shift in dietary structure toward “low-cost, high-carbohydrate” foods, thereby extending the life-cycle hypothesis in the specific context of rural areas. Land management scale, as a mediating variable, reflects the supporting role of agricultural production factors for consumption. For rural aging households with a certain scale of land, land is not only a means of production but also a form of informal security, easing food security anxieties associated with aging and thereby shaping the final composition of food consumption through this mechanism.

This study also has several limitations. First, it uses only cross-sectional data from 2022. The CRRS research team has so far publicly released data from the first wave (CRRS2020) and the second wave (CRRS2022). However, due to revisions to the 2022 questionnaire compared with 2020, some indicators required for this study are only available in the second wave (CRRS2022), which will be officially released in 2025. In addition, the time interval between the two datasets is relatively short, making it difficult to capture changes in the impact of population aging on food consumption. Therefore, this study relies solely on CRRS2022. Although instrumental variables are employed, cross-sectional data cannot, like panel data, use fixed effects to fully eliminate time-invariant, hard-to-observe sources of heterogeneity such as family “cultural genes” and long-term consumption habits. Furthermore, food consumption is deeply shaped by cultural context and individual psychology, realized through market channels, constrained by natural conditions, and guided by macro policies and technologies. This paper only identifies key pathways through which population aging affects rural residents’ food consumption and does not explore other potential channels or interaction effects. Rigorous causal inference still requires future verification based on longer-term, more internally consistent panel data, in order to test the long-run dynamic stability of the impact of aging on food consumption. Future research should incorporate multi-period panel data and comprehensively account for various influencing factors, so as to inform more holistic food consumption strategies.

## 6. Conclusions and Policy Recommendations Discussion

### 6.1. Conclusions

Based on theoretical analysis and empirical testing, this study proposes hypotheses regarding the direct impact of population aging on rural residents’ food consumption and its potential mechanisms. Utilizing micro-household samples from the 2022 data of the “China Rural Revitalization Survey” (CRRS), we empirically investigate the impact of population aging on rural household food consumption. Firstly, this chapter uses the OLS model as the baseline regression model, considering various factors that may influence rural residents’ food consumption, to preliminarily assess the correlation between population aging and food consumption. Secondly, to address potential endogeneity issues in the model, we re-estimate the impact of population aging on rural household food consumption using the instrumental variable method. Subsequently, robustness checks are conducted by replacing the core explanatory variable to verify the regression results. Finally, to explore the mechanisms through which population aging affects food consumption, this study examines the mediating roles of household income level and land management scale. The unique contribution of this study lies in demonstrating that the impact of rural population aging on food consumption is essentially a livelihood reconstruction of the family unit under intensified resource constraints (income and land). This finding enriches the simplistic notion that “aging inevitably leads to consumption contraction,” emphasizing the function of the family as a risk buffer in maintaining energy intake levels, while also highlighting the deep-seated challenges it faces in optimizing dietary structures. The main research conclusions are as follows:

First, the aging population has a significant positive impact on the per capita standard energy intake of rural households. The marginal effects indicate that, after controlling for other factors, a 1 percentage point increase in the share of people aged 60 and above in a household is associated with an average increase of 448.997 kilocalories per day in total energy intake, equivalent to 14.075% of the sample rural residents’ mean daily energy intake. Even after addressing endogeneity and conducting robustness checks, the conclusion that aging raises rural households’ per capita standard total energy intake remains valid. Although aging may lead to reduced consumption of certain foods, the notable increase in grain consumption—due to its high energy density—is sufficient to offset, and even exceed, the energy reductions from other food groups, resulting in an overall upward trend in per capita standard total energy intake. This finding suggests that when examining the impact of population aging on residents’ healthy dietary structure, we need to examine changes in the consumption of various food types more closely and assess their combined effect on total energy intake.

Second, aging affects daily consumption levels of different types of foods differently. Specifically, aging increases per capita standard consumption of grains, vegetables, legumes, dairy products, and eggs, while it suppresses per capita standard consumption of fruits and beef and mutton. Even after addressing endogeneity and conducting robustness checks, the impact of aging on food consumption remains heterogeneous. This structural difference indicates that under the dual pressures of anticipated income decline and the health uncertainties induced by aging, rural households exhibit a clear “economic rationality”: they prioritize low-cost, high-satiation sources of plant-based protein and basic energy, while deliberately reducing higher-price-elastic, premium foods.

Third, household income levels and land management scale serve as important mediating factors in the effect of population aging on food consumption quantities in rural households. As population aging intensifies, it results in decreased household income levels and reduced land management scale, which in turn increases the total energy intake and influences food consumption quantities among rural residents.

### 6.2. Policy Recommendations

Promoting the construction of China’s food monitoring system involves several key steps. First, it is essential to improve monitoring technologies and standards by developing and applying advanced technologies that comprehensively cover all aspects of food production, processing, distribution, and consumption, from the source to the consumer. Second, establishing unified food safety and quality testing standards will ensure the comparability of domestic and international food markets. Finally, conducting scientific research and evaluation is crucial; this includes supporting relevant research institutions to study food safety, nutrition, and health, thereby establishing a direct link between food monitoring and health impacts. Regular assessments of the effectiveness of food monitoring will allow for timely adjustments in strategies and focuses to align with changes in dietary habits.

Improve land transfer and trusteeship mechanisms to offset household factor constraints. On the one hand, invigorate the land transfer and rental market. For households whose land has become fragmented or abandoned due to aging, village-level land transfer service platforms should be established. Through mechanisms such as equity participation with land management rights and land leasing, older adults who have lost labor capacity can obtain stable rental income, thereby mitigating productivity losses caused by physical decline. On the other hand, promote the socialized provision of agricultural services. For older farmers who are “unwilling to transfer land but unable to cultivate it themselves,” full value-chain trusteeship services—such as mechanized ploughing, planting, and harvesting—should be vigorously developed. By substituting machine for manual labor, these services can maintain returns on households’ natural capital, secure basic in-kind provision, and ease livelihood pressures induced by aging.

Broaden income sources for rural residents. First, strengthen the nutritional support function of pensions. Given that pensions are currently used mainly to secure a “subsistence bottom line,” policy should, while steadily raising the basic rural pension level, explore targeted “nutrition subsidies” or “food vouchers” for very old and disabled rural residents, guiding transfer income toward improved food items such as fruits and high-quality protein. Second, empower the rural “silver economy” and flexible employment. Encourage the development of low-intensity, high-value-added sectors suitable for older adults, such as rural e-commerce, agritourism, and handicrafts. Improve rural labor resource databases and provide skills training for older people who are willing to participate, enabling them to share more value-added gains along rural industrial chains and thereby raise the upper bound of the household budget constraint.

Implement comprehensive nutrition and health education. Firstly, strengthen nutrition and health education in rural areas by conducting thorough surveys on local traditional dietary structures, common misconceptions, and key nutritional issues. Transform abstract dietary guidelines into easy-to-understand, practical local languages and examples, respecting and optimizing the healthy elements within traditional diets. Secondly, innovate communication channels and formats, considering the societal characteristics of rural areas and the preferences of rural households. Utilize traditional media channels such as village loudspeakers, bulletin boards, and market activities alongside new media tools like WeChat groups and short video platforms to organize health lectures, cooking demonstrations, and healthy family contests as participatory activities, integrating knowledge dissemination into daily life.

Improve the rural social security system in China. Firstly, gradually increase the basic pension for urban and rural residents, establishing a dynamic adjustment mechanism linked to economic development, price levels, and farmers’ income growth to ensure the actual purchasing power of pensions. Encourage eligible agricultural migrants to participate in basic pension insurance for enterprise employees, providing smooth social security transfer pathways for agricultural migrants working in cities or engaged in flexible employment, thereby expanding the coverage of pension insurance. Secondly, further enhance the basic medical insurance system for urban and rural residents by gradually expanding the reimbursement catalog, increasing the reimbursement ratio and ceilings for major illness insurance, thereby effectively reducing the medical expense burden on elderly farmers. Lastly, steadily raise the social assistance standards for rural subsistence allowances and support for individuals in extreme poverty, ensuring all eligible elderly individuals are included in the coverage.

## Figures and Tables

**Table 1:** List of variables.

**Variables**	**Definitions and Assignments**	**Mean**	**SD**
Explained variable			
Total energy intake	per capita energy intake (kcal) for households	3189.990	1222.947
Grain consumption quantity	per capita grain consumption (grams) for households	491.618	223.931
Vegetable consumption quantity	per capita daily vegetable consumption (grams) for households	357.454	261.819
Fruit consumption quantity	per capita daily fruit consumption (grams) for households	172.864	226.227
Legume consumption quantity	per capita daily legume consumption (grams) for households	24.746	30.025
Dairy product consumption quantity	per capita daily dairy consumption (grams) for households	56.847	105.188
Pork consumption quantity	per capita daily consumption of pork (grams) for households	77.187	66.646
Poultry consumption quantity	per capita daily poultry consumption (grams) for households	32.573	43.252
Beef and lamb consumption quantity	per capita daily consumption of beef and lamb (grams) for households	13.245	30.623
Egg consumption quantity	per capita daily egg consumption (grams) for households	44.314	35.931
Aquatic product consumption quantity	per capita daily Aquatic product consumption (grams) for households	34.543	51.334
Core explanatory variable			
Degree of aging in households	the proportion of the population aged 60 and above in the total household population	0.330	0.383
Mediating variable			
Family income	per capita household income (ten thousand yuan)	9.209	1.532
Land management scale	take the logarithm of the area of land operated by the household	1.672	1.447
Other control variables			
Gender	householder’s gender (1 = male; 0 = female)	0.933	0.250
Ethnicity	householder’s ethnicity (1 = Han; 0 = other)	0.874	0.333
Educational condition	years of education of the householder (years)	8.123	3.039
Marital status	householder’s marital status (1 = married; 0 = other)	0.910	0.286
Political identity	does the household have a party member? (1 = yes; 0 = no).	0.374	0.484
Female proportion	proportion of female family members	0.482	0.190
Medical insurance	proportion of household members who have purchased health insurance	0.970	0.138
Retirement insurance	proportion of household members who have purchased pension insurance	0.693	0.304
Village-county distance	take the logarithm of the distance from the village committee to the county government	2.905	0.780
Economic level	take the logarithm of the per capita annual income of the village	9.450	1.201
Village topography	village terrain (1 = plain; 2 = hilly; 3 = sub-mountainous; 4 = mountainous)	1.939	0.883
Village location	village location (1 = suburban; 2 = non-suburban)	1.811	0.391

**Table 2:** OLS regression of Population Aging and Food Consumption Quantity.

Variables	(1)	(2)	(3)	(4)	(5)	(6)	(7)	(8)	(9)	(10)	(11)
	Energy	Grain	Vegetables	Fruit	Legumes	Dairy	Pork	Poultry	Beef and Lamb	Egg	Aquatic
Aging	448.997 ***	106.857 ***	57.900 ***	−36.316 ***	8.202 ***	9.226 *	0.032	1.036	−5.585 ***	7.116 ***	−3.792
	(63.348)	(11.055)	(13.485)	(11.445)	(1.576)	(5.457)	(3.112)	(2.213)	(1.402)	(1.746)	(2.460)
Gender	15.613	18.113	18.579	−17.318	−2.718	−27.519 ***	−2.301	0.142	−3.996 *	3.202	3.164
	(98.440)	(17.179)	(20.956)	(17.784)	(2.449)	(8.480)	(4.836)	(3.439)	(2.179)	(2.713)	(3.822)
Ethnicity	−100.742	−33.262 **	31.450 *	−12.403	2.082	−9.289	12.352 ***	5.972 **	−5.431 ***	5.383 **	−1.334
	(81.970)	(14.305)	(17.450)	(14.809)	(2.039)	(7.061)	(4.027)	(2.864)	(1.814)	(2.259)	(3.183)
Marital status	−102.761	−16.653	−45.458 **	5.582	−0.980	−0.873	0.008	−1.094	1.514	−5.859 **	3.266
	(85.892)	(14.989)	(18.284)	(15.517)	(2.137)	(7.399)	(4.219)	(3.001)	(1.901)	(2.367)	(3.335)
Education	−11.500	−5.759 ***	0.813	5.137 ***	0.045	1.907 ***	0.547	0.100	0.270	0.255	0.630 **
	(7.888)	(1.377)	(1.679)	(1.425)	(0.196)	(0.680)	(0.388)	(0.276)	(0.175)	(0.217)	(0.306)
Political identity	−8.605	−18.317 **	−9.716	−0.279	1.545	2.686	5.953 **	−1.837	3.913 ***	1.960	4.559 **
	(49.049)	(8.560)	(10.441)	(8.861)	(1.220)	(4.225)	(2.409)	(1.714)	(1.086)	(1.352)	(1.904)
female proportion	−181.150	−11.726	39.747	33.819	−6.343 **	18.165 *	−12.168 *	2.661	−7.943 ***	4.894	−7.476
	(126.553)	(22.085)	(26.940)	(22.863)	(3.148)	(10.901)	(6.217)	(4.421)	(2.801)	(3.488)	(4.914)
Medical insurance	60.366	8.545	30.390	34.869	2.530	4.061	−0.908	12.479 **	4.302	−0.204	7.164
	(163.627)	(28.555)	(34.832)	(29.561)	(4.071)	(14.095)	(8.038)	(5.717)	(3.622)	(4.509)	(6.353)
pension insurance	332.670 ***	43.155 ***	85.395 ***	17.819	3.236	−4.603	13.338 ***	5.507 *	2.772	4.231 *	4.742
	(80.820)	(14.104)	(17.205)	(14.601)	(2.011)	(6.962)	(3.970)	(2.824)	(1.789)	(2.227)	(3.138)
county distance	72.295 **	22.296 ***	−2.849	−0.641	−0.988	−0.330	0.807	0.171	2.083 ***	0.847	−2.556 **
	(31.910)	(5.569)	(6.793)	(5.765)	(0.794)	(2.749)	(1.568)	(1.115)	(0.706)	(0.879)	(1.239)
Economic level	1.572	−0.738	−1.230	−2.346	0.472	0.119	−0.201	−0.336	0.192	0.547	1.016
	(20.193)	(3.524)	(4.299)	(3.648)	(0.502)	(1.739)	(0.992)	(0.706)	(0.447)	(0.556)	(0.784)
topography	29.895	12.303 **	−0.327	−10.946 **	0.867	−1.251	3.958 ***	−5.127 ***	−2.329 ***	−3.405 ***	−4.271 ***
	(29.796)	(5.200)	(6.343)	(5.383)	(0.741)	(2.567)	(1.464)	(1.041)	(0.660)	(0.821)	(1.157)
location	78.269	12.111	16.974	6.600	1.690	−4.749	−0.000	6.169 ***	−0.729	−0.640	−0.145
	(58.628)	(10.231)	(12.481)	(10.592)	(1.459)	(5.050)	(2.880)	(2.048)	(1.298)	(1.616)	(2.276)
constant	2636.102 ***	437.341 ***	232.579 ***	54.735	16.661 **	37.291	37.161 **	−3.613	9.547	33.682 ***	30.278 **
	(323.899)	(56.524)	(68.951)	(58.516)	(8.058)	(27.901)	(15.911)	(11.316)	(7.170)	(8.926)	(12.576)
Provincial fixed effect	YES	YES	YES	YES	YES	YES	YES	YES	YES	YES	YES
Number	2846	2846	2846	2846	2846	2846	2846	2846	2846	2846	2846
R-squared	0.066	0.152	0.077	0.109	0.041	0.063	0.241	0.089	0.270	0.178	0.201

Table notes: *, **, *** denote significance tests at the 10%, 5% and 1% levels, with robust standard errors in parentheses.

**Table 3:** Endogeneity analysis results.

Variables	Total Energy Intake	
	first stage	second stage
Aging		887.628 **
		(406.084)
instrumental variable	0.358 ***	
	(0.043)	
Control variables	YES	
Provincial fixed effect	YES	
first-stage F-statistic	65.312	
Durbin–Wu–Hausman test	1.216	
*p*-value	0.270	
Number	2846	
Wald chi-square value	153.28 ***	
R-squared	0.050	

Table notes: **, *** denote significance tests at the 5% and 1% levels, with robust standard errors in parentheses.

**Table 4:** Robustness analysis.

Variables	(1)	(2)	(3)	(4)	(5)	(6)	(7)	(8)	(9)	(10)	(11)
	Energy	Grain	Vegetables	Fruit	Legumes	Dairy	Pork	Poultry	Beef and Lamb	Egg	Aquatic
Aging	324.939 ***	88.519 ***	50.792 ***	−28.726 **	8.130 ***	18.138 ***	−3.428	2.458	−3.495 **	6.717 ***	−3.668
	(68.562)	(11.987)	(14.541)	(12.337)	(1.699)	(5.870)	(3.351)	(2.383)	(1.513)	(1.882)	(2.649)
Gender	22.383	19.029	18.900	−17.714	−2.730	−28.092 ***	−2.087	0.052	−4.114 *	3.212	3.165
	(98.937)	(17.298)	(20.982)	(17.802)	(2.451)	(8.471)	(4.836)	(3.439)	(2.183)	(2.715)	(3.823)
Ethnicity	−84.726	−30.031 **	33.054 *	−13.571	2.261	−9.555	12.533 ***	5.920 **	−5.659 ***	5.556 **	−1.421
	(82.315)	(14.392)	(17.457)	(14.811)	(2.039)	(7.048)	(4.023)	(2.862)	(1.817)	(2.259)	(3.181)
Marital status	−114.579	−18.276	−46.037 **	6.278	−0.963	0.103	−0.360	−0.939	1.719	−5.879 **	3.267
	(86.367)	(15.100)	(18.317)	(15.540)	(2.140)	(7.395)	(4.221)	(3.002)	(1.906)	(2.370)	(3.337)
Education	−12.871	−5.806 ***	0.858	5.187 ***	0.075	2.165 ***	0.461	0.139	0.301 *	0.272	0.618 **
	(7.997)	(1.398)	(1.696)	(1.439)	(0.198)	(0.685)	(0.391)	(0.278)	(0.176)	(0.219)	(0.309)
Political identity	−13.458	−19.680**	−10.507	0.159	1.416	2.372	6.017 **	−1.880	3.963 ***	1.853	4.617 **
	(49.304)	(8.620)	(10.456)	(8.871)	(1.222)	(4.222)	(2.410)	(1.714)	(1.088)	(1.353)	(1.905)
female proportion	−208.367	−17.844	36.522	35.942	−6.770 **	17.973 *	−12.281 **	2.653	−7.587 ***	4.513	−7.276
	(127.076)	(22.217)	(26.950)	(22.865)	(3.148)	(10.880)	(6.211)	(4.418)	(2.804)	(3.487)	(4.910)
Medical insurance	55.113	7.345	29.752	35.283	2.443	4.005	−0.923	12.475 **	4.370	−0.280	7.204
	(164.421)	(28.747)	(34.870)	(29.585)	(4.074)	(14.078)	(8.036)	(5.716)	(3.629)	(4.512)	(6.354)
pension insurance	413.514 ***	59.312 ***	93.371 ***	11.954	4.108 **	−6.100	14.295 ***	5.223 *	1.616	5.081 **	4.314
	(79.807)	(13.953)	(16.925)	(14.360)	(1.977)	(6.833)	(3.901)	(2.774)	(1.761)	(2.190)	(3.084)
county distance	74.212 **	22.743 ***	−2.609	−0.794	−0.955	−0.299	0.810	0.174	2.059 ***	0.876	−2.572 **
	(32.064)	(5.606)	(6.800)	(5.769)	(0.794)	(2.745)	(1.567)	(1.115)	(0.708)	(0.880)	(1.239)
Economic level	0.412	−1.071	−1.426	−2.240	0.439	0.036	−0.183	−0.347	0.203	0.520	1.030
	(20.294)	(3.548)	(4.304)	(3.652)	(0.503)	(1.738)	(0.992)	(0.705)	(0.448)	(0.557)	(0.784)
topography	31.623	12.914 **	0.054	−11.130 **	0.937	−1.011	3.896 ***	−5.093 ***	−2.340 ***	−3.350 ***	−4.302 ***
	(29.960)	(5.238)	(6.354)	(5.391)	(0.742)	(2.565)	(1.464)	(1.042)	(0.661)	(0.822)	(1.158)
location	77.853	11.712	16.681	6.699	1.624	−5.066	0.092	6.122 ***	−0.738	−0.689	−0.116
	(58.930)	(10.303)	(12.498)	(10.603)	(1.460)	(5.046)	(2.880)	(2.049)	(1.301)	(1.617)	(2.277)
constant	2693.687 ***	449.315 ***	238.630 ***	50.455	17.373 **	36.700	37.699 **	−3.745	8.746	34.352 ***	29.934 **
	(325.290)	(56.872)	(68.987)	(58.530)	(8.059)	(27.852)	(15.899)	(11.308)	(7.179)	(8.927)	(12.570)
Provincial fixed effect	YES	YES	YES	YES	YES	YES	YES	YES	YES	YES	YES
Number	2846	2846	2846	2846	2846	2846	2846	2846	2846	2846	2846
R-squared	0.057	0.140	0.075	0.108	0.040	0.066	0.242	0.089	0.268	0.177	0.201

Table notes: *, **, *** denote significance tests at the 10%, 5% and 1% levels, with robust standard errors in parentheses.

**Table 5:** Mechanism Test Results of the Impact of Population Aging on Consumption Quantity.

Variables	(1)	(2)
	Family Income	Land Management Scale
Aging	−0.407 ***	−0.594 ***
	(0.078)	(0.067)
Control variable	YES	YES
Provincial fixed effect	YES	YES
Number	2846	2846
R-squared	0.092	0.250

Table notes: *** denote significance tests at the 1% levels, with robust standard errors in parentheses.

## Data Availability

The datasets presented in this article are not readily available because this is non-public data. Requests to access the datasets should be directed to the Rural Development Institute, Chinese Academy of Social Sciences.
